# Prefrontal cortex activity during binocular color fusion and rivalry: an fNIRS study

**DOI:** 10.3389/fneur.2025.1527434

**Published:** 2025-04-14

**Authors:** Xiang Liu, Xuesong Jin, Lijun Yun, Zaiqing Chen

**Affiliations:** ^1^Yunnan Key Laboratory of Optoelectronic Information Technology, Kunming, China; ^2^Yuxi Key Laboratory of Mental Health Examination, Yuxi, Yunnan, China; ^3^Department of Education of Yunnan Province, Engineering Research Center of Computer Vision and Intelligent Control Technology, Kunming, China; ^4^School of Information Science and Technology, Yunnan Normal University, Kunming, China

**Keywords:** binocular color fusion and rivalry, functional near-infrared spectroscopy (fNIRS), prefrontal cortex (PFC), brain functional connectivity, generalized linear model

## Abstract

**Introduction:**

Understanding how the brain processes color information from both the left and right eyes is a significant topic in neuroscience. Binocular color fusion and rivalry, which involve advanced cognitive functions in the prefrontal cortex (PFC), provide a unique perspective for exploring brain activity.

**Methods:**

This study used functional near-infrared spectroscopy (fNIRS) to examine PFC activity during binocular color fusion and rivalry conditions. The study included two fNIRS experiments: Experiment 1 employed long-duration (90 s) stimulation to assess brain functional connectivity, while Experiment 2 used short-duration (10 s) repeated stimulation (eight trials), analyzed with a generalized linear model to evaluate brain activation levels. Statistical tests were then conducted to compare the differences in brain functional connectivity strength and activation levels.

**Results:**

The results indicated that functional connectivity strength was significantly higher during the color fusion condition than the color rivalry condition, and the color rivalry condition was stronger than the Mid-Gray field condition. Additionally, brain activation levels during binocular color fusion were significantly greater, with significant differences concentrated in channel (CH) 12, CH13, and CH14. CH12 is located in the dorsolateral prefrontal cortex, while CH13 and CH14 are in the frontal eye fields, areas associated with higher cognitive functions and visual attention.

**Discussion:**

These findings suggest that binocular color fusion requires stronger brain integration and higher brain activation levels. Overall, this study demonstrates that color fusion is more cognitively challenging than color rivalry, engaging more attention and executive functions. These results provide theoretical support for the development of color-based brain-computer interfaces and offer new insights into future research on the brain's color-visual information processing mechanisms.

## 1 Introduction

When the left and right eyes are presented with different colors, such as red and green, and if the color difference is minimal, the brain can integrate these two colors into a unified perception, a phenomenon known as binocular color fusion (1). Conversely, when the color difference exceeds a certain threshold, the brain perceives an alternating sequence of the two colors, a phenomenon known as binocular color rivalry (2, 3). These two objective stimulus conditions generate completely distinct and independent subjective perceptions, with no transformation between them in terms of experience. During binocular color fusion, the observer perceives a stable and unified single-color experience (4). In contrast, binocular color rivalry is characterized by the dynamic alternation of two colors at a specific frequency (5). These two perceptual modes involve distinct processing mechanisms, and their subjective experiences consistently retain their unique characteristics. Human vision achieves a unified experience through binocular collaboration. Modern stereoscopic display technology creates depth perception by presenting different perspective images to each eye. However, significant color differences may trigger color fusion or rivalry, potentially affecting display quality and visual comfort. Therefore, understanding the mechanisms of binocular color processing is essential for analyzing visual information integration and conflict resolution.

In the study of binocular color fusion and rivalry mechanisms, many unanswered questions remain. Some studies focus on the perceptual features of binocular vision but lack an in-depth exploration of the underlying neural mechanisms. For example, Hu et al. (6) studied the effect of luminance on the limits of binocular color fusion, while Klink and Roelfsema (7) investigated binocular rivalry under unconscious conditions. However, these studies have not addressed higher cognitive regions. The processes of binocular color fusion and rivalry likely involve the prefrontal cortex (PFC), particularly when processing conflicting color stimuli. The PFC, as a critical region responsible for integrating conflicting information and regulating attention, may coordinate inputs from both eyes through its functions in conflict monitoring and decision-making to achieve stable perception (8, 9). As a core region for high-level cognitive functions, the PFC plays a crucial role in attention control, decision-making, and conflict monitoring (9). Moreover, the PFC is essential for managing conflicting information and regulating perceptual selectivity (10, 11). Investigating the role of the PFC not only enhances our understanding of the neural mechanisms that regulate visual conflicts but also provides theoretical support for improving visual comfort in stereoscopic display technology. However, direct evidence of how the PFC regulates conflicts in binocular color fusion and rivalry remains lacking (12). Identifying core functional regions of the PFC that differentiate binocular color fusion from rivalry and exploring how the PFC participates in decision-making and conflict resolution is crucial. Functional characteristics and roles in binocular color fusion and rivalry need further research.

Current neuroscience research primarily uses tools such as functional magnetic resonance imaging (fMRI). However, due to equipment limitations, fMRI is difficult to use with metallic devices. Functional near-infrared spectroscopy (fNIRS) is a non-invasive brain imaging technology that can monitor hemodynamic changes in the cerebral cortex in real time, providing high spatial resolution and offering portability (13, 14). fNIRS has been widely used to study brain functions during various cognitive tasks (15, 16). For example, Wu et al. (17) used fNIRS to explore how different visual cues affect cortical activation and functional connectivity, showing significant effects on hemodynamic responses in the cortex. Ren et al. (18) found that fNIRS effectively reflects prefrontal activation patterns during different cognitive tasks. Cai et al. (19) used fNIRS to study cortical neural correlates of visual fatigue during binocular depth perception. Although fNIRS has been widely applied in visual perception and cognitive function research, few studies have specifically used fNIRS to investigate binocular color fusion and rivalry.

**Figure 1:**
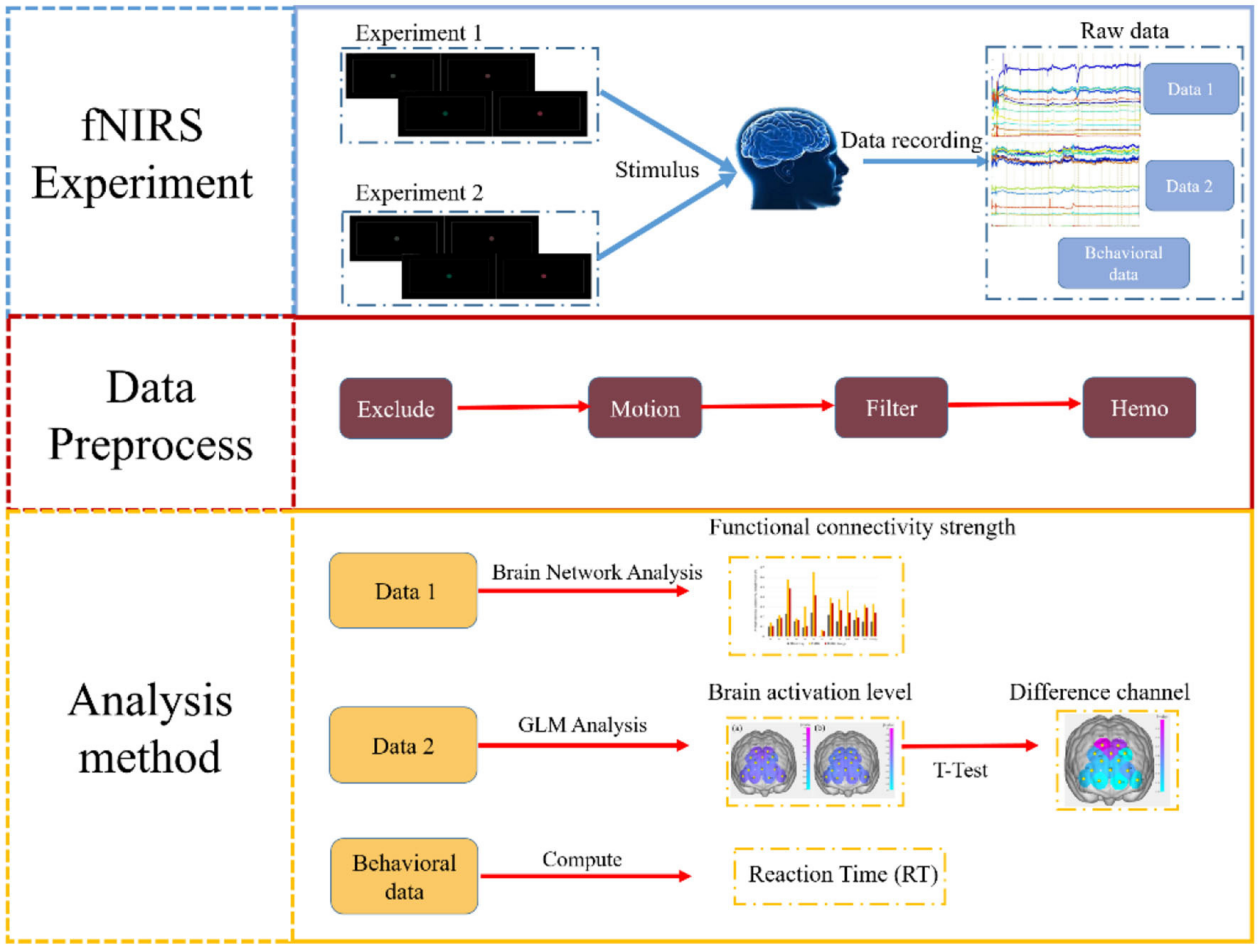
Flowchart of the experiment, data preprocessing, and analysis methods. The experimental design includes two experiments, referred to as Experiment 1 and Experiment 2. The data preprocessing consists of four main steps. The analysis methods primarily include brain network analysis and GLM analysis.

In this study, we utilized a 14-channel fNIRS system arranged according to the international 10–20 system. The brain regions corresponding to these channels were determined based on Brodmann Areas (BAs) (20), with the mappings shown in Table 1. To explore the functional characteristics of the PFC under binocular color fusion and rivalry stimuli, we designed two fNIRS experiments. Experiment 1 aimed to investigate the functional connectivity characteristics of the PFC under these stimuli, while Experiment 2 focused on identifying key brain regions that distinguish binocular color fusion from rivalry through activation analysis. The channels were distributed across various locations in the PFC, and analyzing these channels helped identify critical brain regions associated with fusion and rivalry. Additionally, reaction times (RT) were calculated from behavioral data to verify whether participants accurately completed the tasks in Experiment 2. The study framework is illustrated in Figure 1. This study provides an in-depth exploration of the functional connectivity characteristics of binocular color fusion and rivalry using fNIRS, addressing a gap in the field of visual cognitive neuroscience. The findings not only deepen our understanding of the neural mechanisms underlying binocular vision and visual conflicts but also provide critical theoretical support for improving stereoscopic display technology and advancing color vision-based brain-computer interface (BCI) technology.

**Table 1:** Brodmann areas corresponding to channels.

**Channels**	**Brodmann Area (Chris rorden' MRIcro)**
CH2, CH3, CH4, CH5, CH6, CH9	Frontopolar area
CH1, CH7, CH8, CH10, CH11, CH12	Dorsolateral prefrontal cortex
CH13, CH14	Includes frontal eye fields

## 2 Materials and methods

### 2.1 Equipment and experimental environment

**Figure 2:**
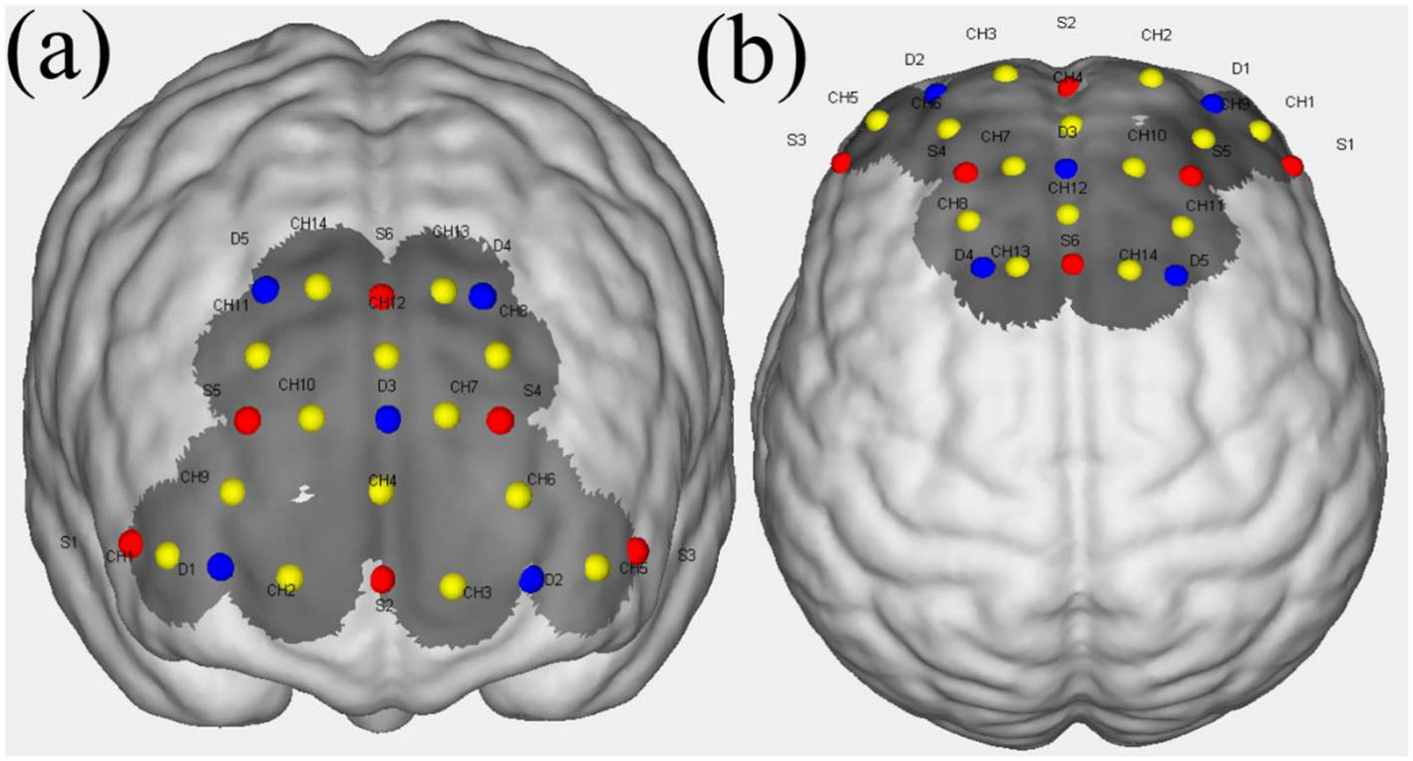
Distribution of light sources, signal detectors, and channels. Red points represent the emission light sources, blue points represent the light sensors, yellow points represent the channels, and the dark-colored area represents the detectable region. **(a)** Front view, **(b)** top view.

This experiment utilized the equipment (model: NirSmart-3000K) from HuiChuang Medical. The equipment comprises a head cap with 14 channels, a functional imaging module, and a computer with NirSmart and NirSpark software. The device has a sampling rate of 20 Hz, features 730 nm and 850 nm spectral illumination, and provides high-precision brain oxygen analysis. Figure 2 illustrates the spatial distribution of light sources, light sensors, and channels in the 3D brain model. In an fNIRS system, a channel refers to the region between a pair of light sources and light sensors. The light emitted by the source passes through the cortical tissue and is then received by the light sensor, and this entire process is referred to as the measurement of one channel. In the figure, red raised points represent the emission light sources, blue raised points represent the light sensors, yellow raised points represent the channels, and the dark-colored area represents the detectable region. There are six light sources (S), 5 light sensors (D), and 14 channels. The arrangement of the channels follows the international standard 10–20 system.

The experimental protocol was implemented via a computer with E-prime software, providing stimulus images and event markers to the NirSmart software. Stimuli were presented on a 23-inch Samsung 3D monitor (model: S23A950D) with a resolution of 1,920 × 1,080 pixels, featuring 2D/3D switching functionality and paired with 3D glasses. The display was connected to an NVIDIA GeForce GTX 1080 graphics card. We used a PR-715 spectroradiometer to measure and calibrate the displayed colors. The lookup table (LUT) method was used to obtain brightness values corresponding to digital input and the luminance and chromaticity of the display's center point. The CIE XYZ values of the black point of the 3D display were 0.247/0.241/0.467, the CIE-1931 chromaticity (x, y) of the white point was (0.274, 0.280), and the chromaticities (x, y) of red, green and blue channels were (0.617, 0.333), (0.330, 0.616) and (0.152, 0.067), respectively.

**Figure 3:**
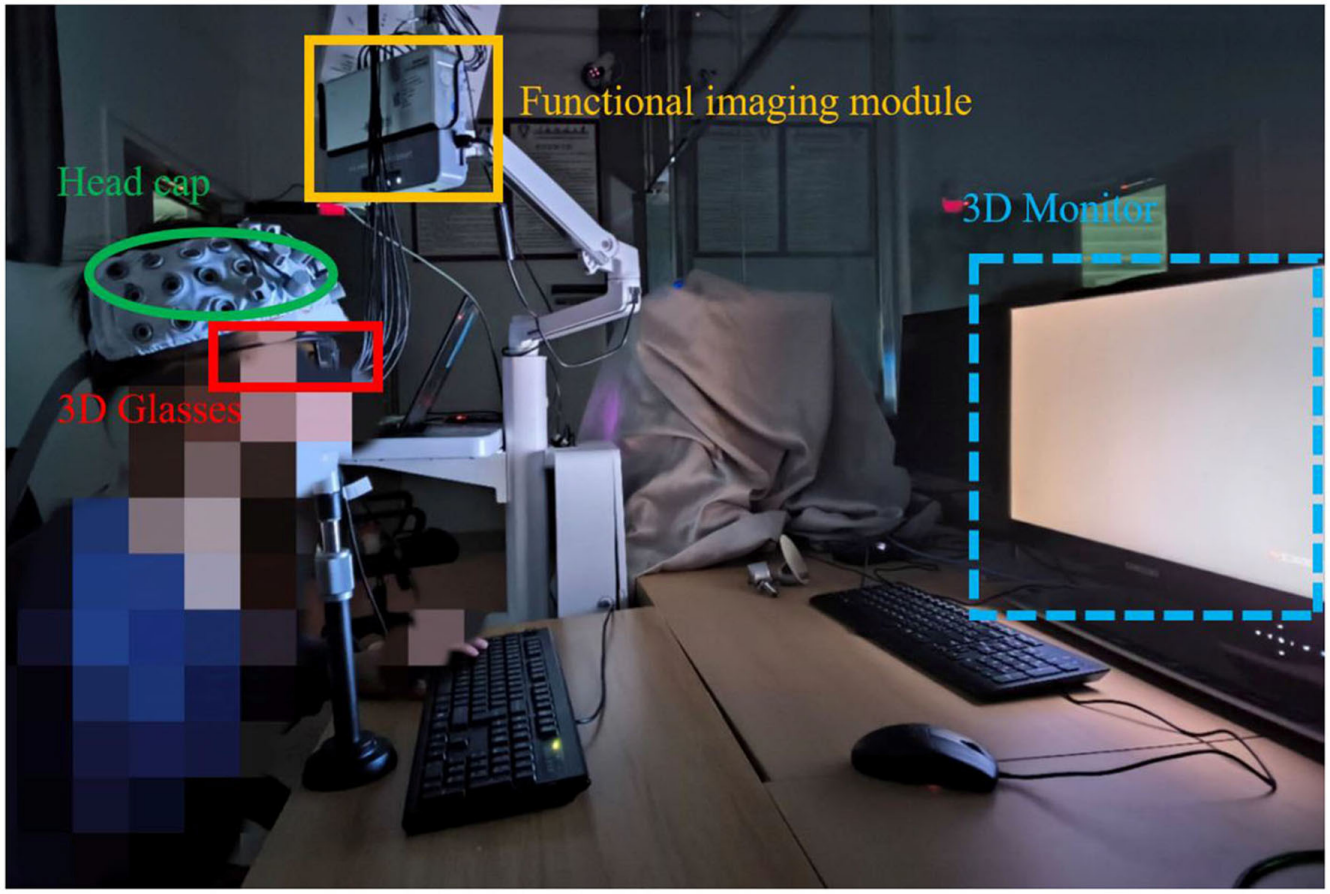
Experimental equipment. The participants wore head caps and 3D glasses while observing stimulus images through a 3D monitor.

The experiment was conducted in a darkroom to minimize other factors (mainly ambient light). Participants were instructed to minimize physical movement during the experiment to reduce motion artifacts that could affect the near-infrared imaging data. According to the International Telecommunication Union standard (38) participants sit approximately 860 millimeters from the screen. Figure 3 shows the experimental setup and environment.

### 2.2 Participants

Twelve graduate students were recruited as participants for this experiment, with an age range of 24–26 years and mean age of 25 years. All participants had normal color vision (assessed using Dvorine Color Plates, 2nd ed.), normal stereoscopic vision (tested with Random Dot Stereoacuity), and normal or corrected visual acuity. They were all non-experts and had not worked with stereo vision. Before the experiment began, each participant signed an informed consent form, which adhered to the ethical standards of the Declaration of Helsinki (21).

### 2.3 Stimuli

**Figure 4:**
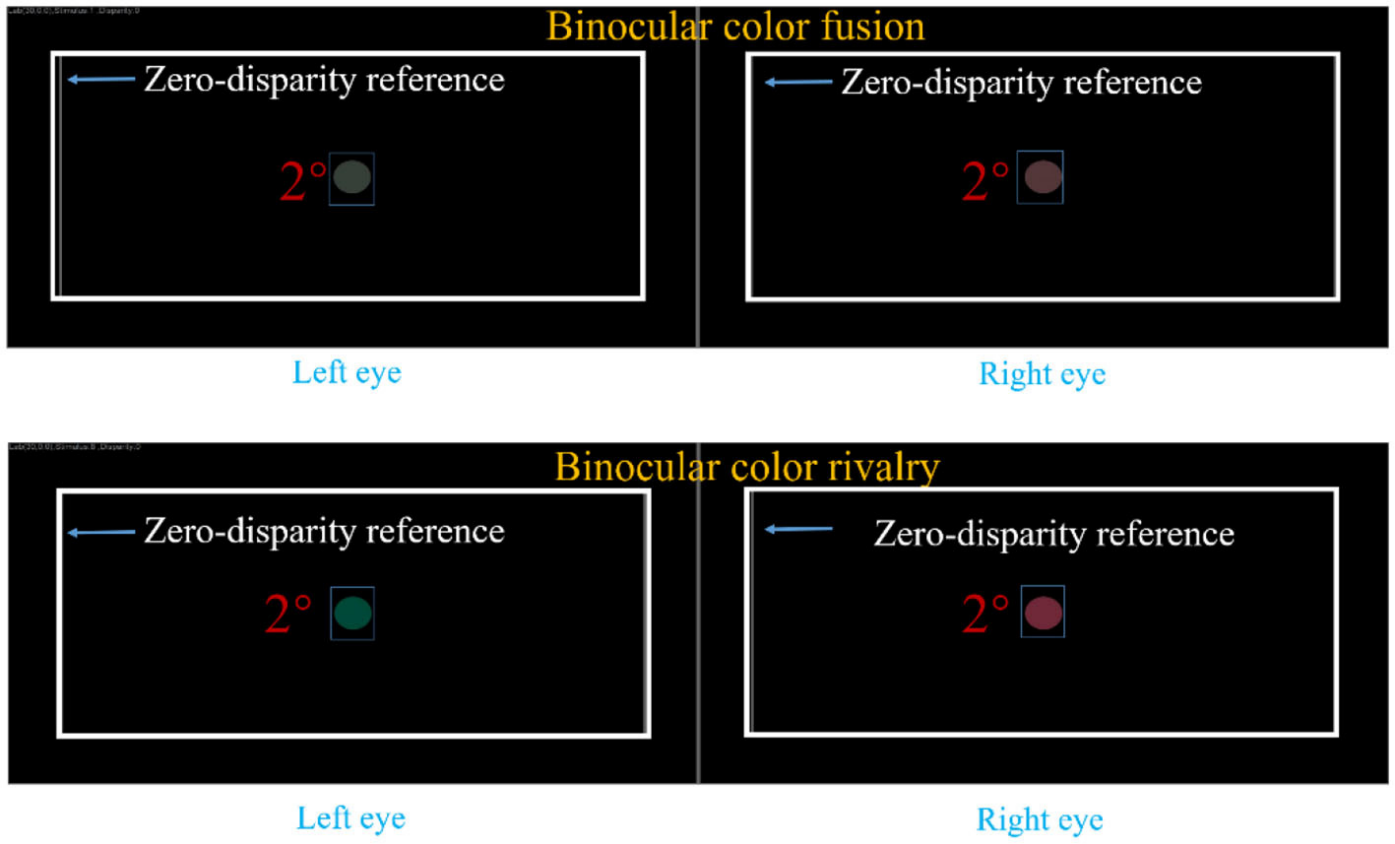
Color stimulus samples. The stimulus images had a resolution of 3,840 × 1,080 pixels, with each image containing a green 1,920 × 1,080 pixel image and a red 1,920 × 1,080 pixel image. These images consisted of a centrally displayed colored circular patch with a visual angle of 2° on a black background.

The stimulus images were generated using specially developed C++ software (Figure 4). The generated images have 3,840 × 1,080 pixels, with participants wearing 3D glasses to receive images of 1,920 × 1,080 pixels per eye (left and right). These images consisted of a centrally displayed colored circular patch with a visual angle of 2° on a black background. The colors of patches were selected from the CIELAB color space, with the luminance fixed at L^*^ = 30. The color values for the circular patches were chosen along the a^*^ (red-green) axis, and the specific values are provided in Table 2. The color remains constant during the presentation, with the color input to the left eye always being green and the color input to the right eye always being red. The selection of these color samples is based on previous research by Xiong et al. (22), which demonstrated that these color combinations effectively induce binocular color fusion and rivalry in most individuals with normal vision. Based on the different color values selected along the red/green axis, two types of stimulus images were created: binocular color fusion on red/green direction (FoRG) and binocular color rivalry on red/green direction (RoRG). FoRG and RoRG can be considered as two objective conditions. When the left and right eyes receive different colors, a larger color difference induces binocular color rivalry, while a smaller color difference induces binocular color fusion. To minimize or eliminate the effects of disparity cues, a gray rectangular frame was added to the stimulus images as a zero-disparity reference, based on the study by Chen et al. (23). Disparity cues can evoke additional depth perception, which may alter the brain's integration of binocular images and, in turn, affect the perception of color fusion or rivalry. Since participants might experience potential small disparities due to equipment calibration or the characteristics of the visual stimuli, we provided a zero-disparity reference to avoid interference with the results. The zero-disparity reference ensures the spatial alignment of the images presented to both eyes, reducing or eliminating the potential effects of disparity cues on the perception of color fusion and rivalry.

**Table 2:** Color stimulation values in the CIELAB color space (FoRG indicates binocular color fusion on red/green direction, and RoRG indicates binocular color rivalry on red/green direction).

**Color stimulation type**	**Sample points pair**			
	**Left eye**		**Right eye**	
	a^*^	b^*^	a^*^	b^*^
FoRG	−9	0	9	0
RoRG	−24	0	24	0

a^*^ (red-green), and b^*^ (yellow-blue).

### 2.4 Procedure

**Figure 5:**
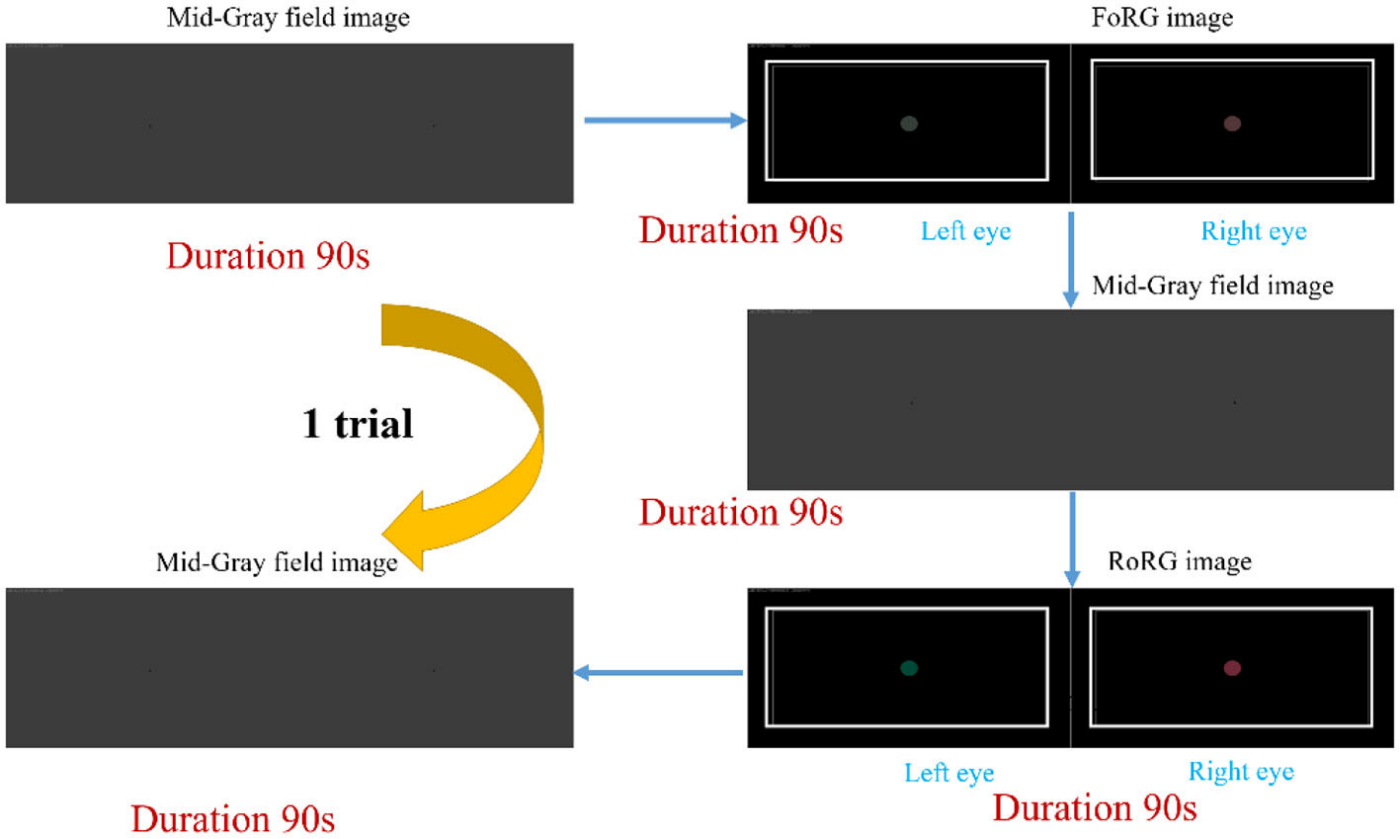
The presentation sequence for experiment 1. Experiment 1 consisted of a single trial, with each image presented only once for a duration of 90 s. The presentation sequence was as follows: (1) Mid-Gray field, (2) FoRG, (3) Mid-Gray field, (4) RoRG, (5) Mid-Gray field.

Each participant underwent one session of experiment 1 and experiment 2. Each session did not exceed 15 min, including explanations and instructions given to the participants. In the experiments, a Mid-Gray field image was used to mitigate visual aftereffects and reduce visual fatigue. The procedure for experiment 1 was as follows: (1) Present a 90-s Mid-Gray field image; (2) Present a 90-s FoRG image; (3) Present a 90-s Mid-Gray field image; (4) Present a 90-s RoRG image; (5) Present another 90-s Mid-Gray field image. The purpose of Experiment 1 was to study the overall functional connectivity strength of the PFC under three conditions: no binocular color stimulus (gray field), binocular color fusion, and binocular rivalry. Geng et al. (24) found that functional connectivity strength stabilizes and becomes reproducible in fNIRS signals after 1 min of data collection. Therefore, we chose a longer stimulus presentation duration (90 s) and presented each stimulus only once to avoid any potential impact on data reliability due to insufficient stimulus presentation time. During the experiment, participants were instructed to sit still in a chair and focus on the center of the screen to ensure they received the physical stimuli presented by the 3D display monitor. Figure 5 illustrates the stimulus presentation sequence for experiment 1.

**Figure 6:**
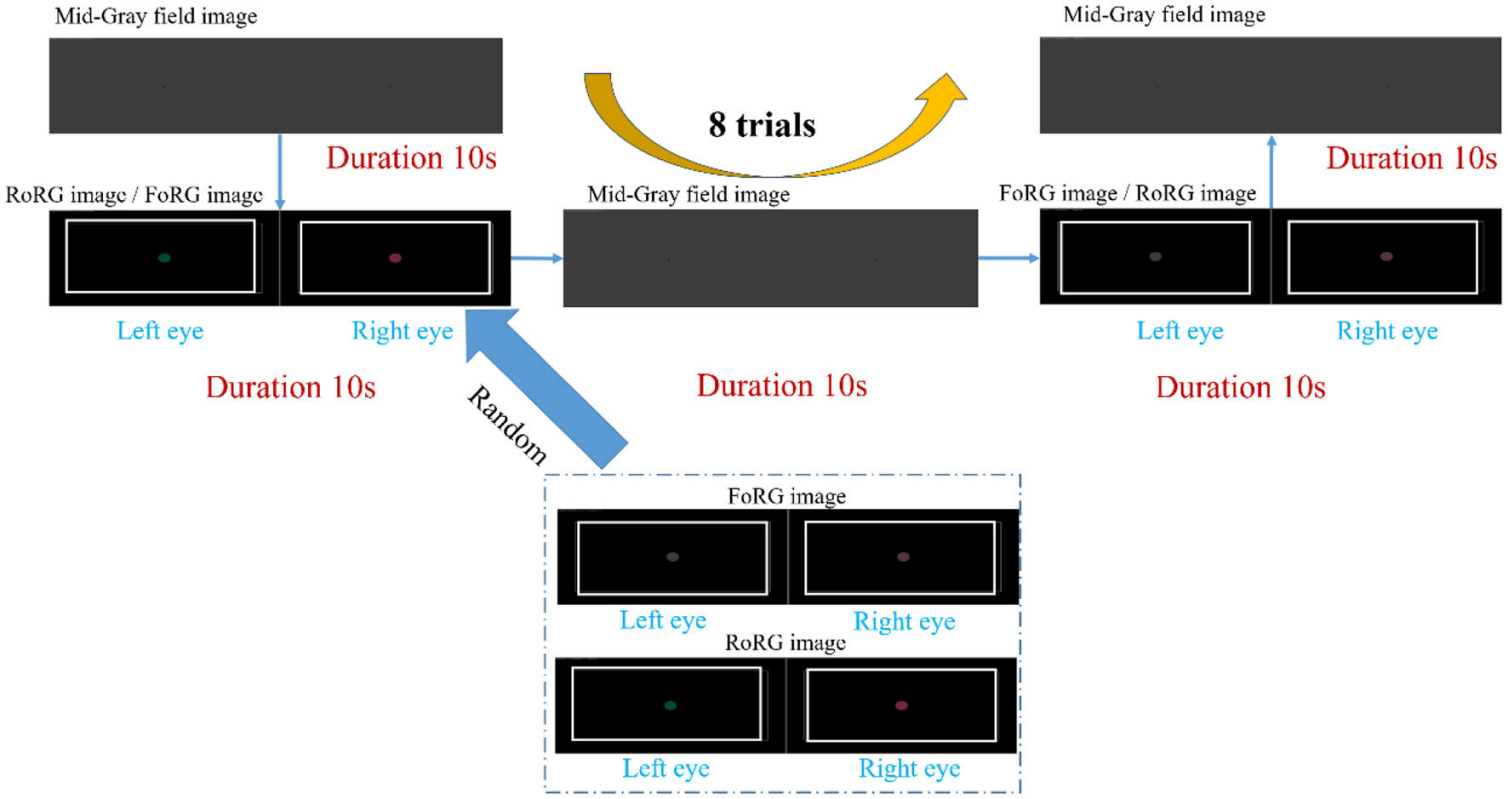
Stimulus presentation sequence for experiment 2. Experiment 2 consisted of 8 trials, each following the sequence below: (1) presentation of a Mid-Gray field image for 10 seconds; (2) random presentation of FoRG image/RoRG image for 10 s; (3) presentation of a Mid-Gray field image for 10 s; (4) presentation of FoRG image/RoRG image for 10 s (if FoRG was presented in step 2, RoRG is presented this time, and vice versa); (5) presentation of a Mid-Gray field image for 10 s. When participants perceived binocular color rivalry, they were instructed to press the “A” key on the keyboard.

Experiment 2 consisted of eight trials, each following the sequence below: (1) Presentation of a Mid-Gray field image for 10 s; (2) Random presentation of FoRG image/RoRG image for 10 s; (3) Presentation of a Mid-Gray field image for 10 s; (4) Presentation of FoRG image/RoRG image for 10 s (if FoRG was presented in step 2, RoRG is presented this time, and vice versa); (5) Presentation of a Mid-Gray field image for 10 s. The focus of Experiment 2 was to identify key regions that distinguish between color fusion and rivalry, which required faster repeated trials to capture more diverse brain activity patterns. The duration of each stimulus presentation was set to 10 s in a similar study (25). Thus, we chose a shorter stimulus presentation duration (10 s) with each stimulus repeated eight times to maximize data collection efficiency while minimizing the potential for visual fatigue and other nonspecific effects caused by prolonged stimulation. For this experiment, data were collected during the presentation of the stimulus images (FoRG and RoRG). Each of the 12 participants underwent this experiment once. During the experiment, participants were instructed to sit in a chair and focus on the center of the screen. When participants perceived binocular color rivalry, they were asked to press the “A” key on the keyboard. After pressing “A,” the stimulus continued to be presented until the end of the 10-s stimulus duration. Simultaneously, the data acquisition software Nirsmart recorded a tag (value = 1). This tag was used to calculate the participants' reaction time (RT), aiding in the collection of behavioral data. By analyzing the reaction time, we can further investigate the behavioral characteristics of binocular color fusion and integrate these data with brain activity data (fNIRS signals) to gain deeper insights into the neural mechanisms of binocular visual phenomena. Figure 6 illustrates the stimulus presentation sequence for experiment 2.

### 2.5 Data preprocess

We performed four steps of preprocessing on the collected fNIRS data:

1. Exclude: Specific time intervals were removed from the dataset, particularly segments outside the stimulus presentation periods or those displaying significant noise artifacts. This step ensured that only relevant, high-quality data were retained for analysis, minimizing the impact of external fluctuations.
2. Motion: We employed a moving standard deviation and spline interpolation method to eliminate motion artifacts. The standard deviation threshold (std_thr) was set to 6. For light intensity data, if the difference between the maximum and minimum values within a 0.5-s time window exceeded six times the mean, that time window was identified as containing motion artifacts and was excluded. Similarly, for optical density, the amplitude threshold (amp_thr) was set to 0.5; if the difference between the highest and lowest values of optical density exceeded 0.5, the data were considered to contain motion artifacts and were removed. This method aligns with recent studies that have used similar techniques to mitigate motion-related noise in fNIRS data.
3. Filter: A band-pass filter was applied, with a high-pass cutoff frequency of 0.01 Hz and a low-pass cutoff frequency of 0.2 Hz. The high-pass filter was used to remove low-frequency drifts unrelated to the experimental data, such as slow changes in instrument response or baseline drift. The low-pass filter was used to eliminate high-frequency noise from the instrument and physiological noise introduced by heartbeat or respiration. These filtering parameters adhere to standard practices in fNIRS data processing.
4. Hemo: The final step involved converting the preprocessed raw data into changes in hemoglobin concentration using the modified Beer-Lambert law, with the Differential Pathlength Factor (DPF) set to 6. This conversion is crucial for interpreting optical measurements in terms of cerebral hemodynamics.

By systematically applying these preprocessing steps, we improved the quality of the fNIRS data, ensuring the reliability of subsequent analyses. All of the preprocessing steps mentioned above were carried out using the NirSpark software. Raw data can be downloaded at: https://doi.org/10.6084/m9.figshare.27330858.

### 2.6 Brain network analysis

The data from Experiment 1 was primarily used for brain network analysis. Functional Connectivity (FC) is an important measure used to evaluate the temporal coordination of activities between different brain regions. Unlike anatomical connectivity, FC reflects functional relationships, providing insights into the interactions between various brain areas. Studying FC is essential for understanding the brain's functional organization, modes of information processing, and neural mechanisms under different tasks or stimuli. Changes in functional connectivity patterns have been associated with cognitive processes and neurological disorders (26).

The Pearson correlation coefficient is a commonly used method for quantifying the linear relationship between two time series (27). Functional connectivity analysis is typically used to calculate the correlation between blood oxygen level-dependent signals from different brain regions, thereby assessing the synchrony and strength of functional connectivity (28). This method has also been successfully applied in fNIRS research to construct functional connectivity networks.

Given the time series data of two brain regions *X* = {*x*_1_, *x*_2_, …, *x*_*n*_} and *Y* = {*y*_1_, *y*_2_, …, *y*_*n*_}, the Pearson correlation coefficient *r*_*XY*_ is calculated as:


(1) $$\begin{array}{l} r_{XY}=\frac{\overset{n}{\underset{i\;=\;1}{\sum }}\left(x_{i}\;-\;\bar{X}\right)\left(y_{i}\;-\;\bar{Y}\right)}{\sqrt{\overset{n}{\underset{i\;=\;1}{\sum }}{\left(x_{i}\;-\;\bar{X}\right)}^{2}}\sqrt{\overset{n}{\underset{i\;=\;1}{\sum }}{\left(y_{i}\;-\;\bar{Y}\right)}^{2}}} \end{array}$$

Where $\overset{n}{\underset{i=1}{\sum }}\left(x_{i}-\;\bar{X}\right)\left(y_{i}-\;\bar{Y}\right)$ is the covariance between *X* and *Y*, reflecting the extent to which both signals deviate from their respective means simultaneously.$\sqrt{\overset{n}{\underset{i=1}{\sum }}{\left(x_{i}\;-\;\bar{X}\right)}^{2}}$ and $\sqrt{\overset{n}{\underset{i=1}{\sum }}{\left(y_{i}-\bar{Y}\right)}^{2}}$ are the standard deviations of *X* and *Y*, respectively, used to normalize the covariance. By calculating the Pearson correlation coefficients between all channels, we can construct a functional connectivity matrix *R*:


(2) $$\begin{array}{l} R=\left(\begin{array}{cccc} r_{11} & r_{12} & \cdots & r_{1n}\\ r_{21} & r_{22} & \cdots & r_{2n}\\ \vdots & \vdots & \ddots & \vdots \\ r_{n1} & r_{n2} & \cdots & r_{nn} \end{array}\right) \end{array}$$

In Equation 3, *r*_*ij*_ represents the functional connectivity strength between the *i*−*th* and *j*−*th* channels. Sum all the selected correlation coefficients *r*_*ij*_ and divide by the number of coefficients *N* to obtain the mean functional connectivity strength $\bar{r}$ :


(3) $$\begin{array}{l} \bar{r}=\frac{1}{N}\underset{i\neq j}{\sum }\;r_{ij} \end{array}$$

### 2.7 GLM analysis

The data from experiment 2 were analyzed using a generalized linear model (GLM). GLM is a statistical method widely used to analyze fNIRS and fMRI data. Its core principle lies in constructing a design matrix to describe the effect of experimental stimuli on brain function signals and using a regression model to estimate the neural responses under different experimental conditions. By incorporating a standard Hemodynamic Response Function (HRF), GLM not only captures signal changes due to neural activity but also reflects the time-delay characteristics of the hemodynamic response, thereby providing a more accurate depiction of the physiological reactions of the brain to specific stimuli. The basic formula for GLM is as follows:


(4) $$\begin{array}{l} Y=X\text{β}+\varepsilon \end{array}$$

Where*Y* represents the observed fNIRS signal (changes in oxyhemoglobin concentration). *X* is the design matrix, which includes the experimental stimulus events and other potential confounding factors. β represents the regression coefficients to be estimated, which indicate the response strength of the brain under different experimental conditions. ε denotes the residuals, representing unexplained noise or error.

The GLM involves convolving the design matrix X with a standard HRF to describe the brain's response to stimuli accurately. The HRF is a function that describes the hemodynamic response following neural activity and is often modeled as a double gamma function:


(5) $$\begin{array}{l} h\left(t\right)=\left(\frac{t^{a_{1}\;-\;1}e^{-\frac{t}{b_{1}}}}{{\left(b_{1}\right)}^{a_{1}}\text{ Γ }\left(a_{1}\right)}\right)\;-c\;\left(\frac{t^{a_{2}\;-\;1}e^{-\frac{t}{b_{2}}}}{{\left(b_{2}\right)}^{a_{2}}\text{ Γ }\left(a_{2}\right)}\right) \end{array}$$

In Equation 5, *t* represents time, starting from the point when the stimulus occurs. *a*_1_ and *b*_1_ are the shape and scale parameters for the positive peak, which describe the initial increase in blood flow following neural activity. *a*_2_ and *b*_2_ are the shape and scale parameters for the negative peak, which describe the dip during the recovery phase of blood flow. *c* is the scaling factor that controls the amplitude of the negative peak. Γ(*a*) is the gamma function used for normalization purposes.

### 2.8 Statistical analysis

To control the false positive rate caused by multiple comparisons, this study adopted the False Discovery Rate (FDR) correction method. FDR correction dynamically adjusts the significance threshold to effectively control the false discovery rate (i.e., the proportion of false positives among all significant results), making it particularly suitable for multi-channel and multi-condition brain imaging studies. The Benjamini-Hochberg method was used to correct all *p*-values in this study. The specific steps include: first, all *p*-values are sorted in ascending order; second, for each test, a critical value is calculated based on the significance level α (set to 0.05), the total number of tests *m*, and the rank *i*:


(6) $$\begin{array}{l} p_{critical\;}=\frac{i}{m}\cdot \alpha \end{array}$$

Then, the largest *p*-value *p*_(*i*)_ that satisfies *p*_(*i*)_ ≤ *p*_*critical*_ is identified, and this *p*_(*i*)_ and all smaller *p*-values are considered significant. Compared to the traditional Bonferroni correction, FDR correction is more flexible, effectively controls the false discovery rate, and retains higher statistical power, making it suitable for large-scale multiple comparison studies.

The Friedman test is a non-parametric method used to analyze the differences among three or more conditions in repeated measures data. It serves as a non-parametric alternative to repeated measures ANOVA, particularly suitable for cases where the data do not follow a normal distribution or contain outliers. The principle of the Friedman test is based on analyzing ranked data instead of directly using the raw data. For each subject, the measurements under multiple conditions are ranked, and the ranks are summed. These rank sums are then compared to determine whether significant differences exist among the conditions. The formula for calculating its test statistic is:


(7) $$\begin{array}{l} \chi_{F}^{2}=\frac{12}{n\cdot k\cdot \left(k+1\right)}\overset{k}{\underset{j=1}{\sum }}R_{j}^{2}-3n\left(k+1\right) \end{array}$$

*n* represents the number of subjects, *k* is the number of conditions, *R*_*j*_ is the rank sum for condition *j*. The null hypothesis (H_0_) assumes that the median values across all conditions are equal. By calculating the test statistic $\chi_{F}^{2}$ and comparing it to the chi-squared distribution with *k*−1 degrees of freedom, the significance level (*P*-value) is determined. If the *P*-value is less than the chosen significance level (0.05), the null hypothesis is rejected, indicating significant differences among the conditions.

The Wilcoxon Signed-Rank Test is a non-parametric statistical method used to compare the differences between two paired datasets. Compared to the paired *t*-test, the Wilcoxon test does not require assumptions about data distribution, making it particularly suitable for small sample sizes or data that deviate from normality, and it is more robust to outliers. The test procedure includes: first, calculating the differences between paired data *d*_*i*_ = *X*_*i*_−*Y*_*i*_; then, ranking the absolute values of the differences and assigning ranks (Ranks), followed by calculating the positive rank sum *W*^+^ and negative rank sum *W*^−^ based on the sign of the original differences. The test statistic *W* is defined as the smaller of the two rank sums:


(8) $$\begin{array}{l} W=\min\left(W^{+},W^{-}\right) \end{array}$$

Based on *W* and the sample size *n*, the *p*-value is calculated to determine significance. In Experiment 1, the Wilcoxon Signed-Rank Test was used to compare brain functional connectivity strengths across the Mid-Gray, FoRG, and RoRG conditions; in Experiment 2, it was applied to compare the GLM results of the same channels (a total of 14 channels) under the FoRG and RoRG conditions.

To evaluate the statistical capability of the experimental design with the current sample size, this study conducted a *post-hoc* power analysis. Power analysis is used to assess the ability of a statistical test to correctly reject the null hypothesis (H0) when a true effect exists. After applying the FDR correction, the significance level for *post-hoc* power analysis can remain at 0.05 because the purpose of power analysis is to evaluate the capability of the statistical test rather than to control the false positive rate in multiple comparisons. The effect size is calculated using Cohen's *d* formula:


(9) $$\begin{array}{l} \text{d}=\frac{\text{Mean Difference }}{\text{ Standard Deviation of Differences}} \end{array}$$

Where Mean Difference represents the mean difference between two conditions, and Standard Deviation represents the standard deviation of the differences. The formula for Power is:


(10) $$\begin{array}{l} \text{Power }=1-\beta \end{array}$$

β represents the probability of a Type II error, which is the failure to detect a true effect (failure to reject H_0_). In this study, the statistical power was calculated based on the sample size (12 participants), a significance level (α = 0.05), and the effect size (d).

The statistical analysis in this study was conducted using Python.

## 3 Results

### 3.1 Functional connectivity

**Figure 7:**
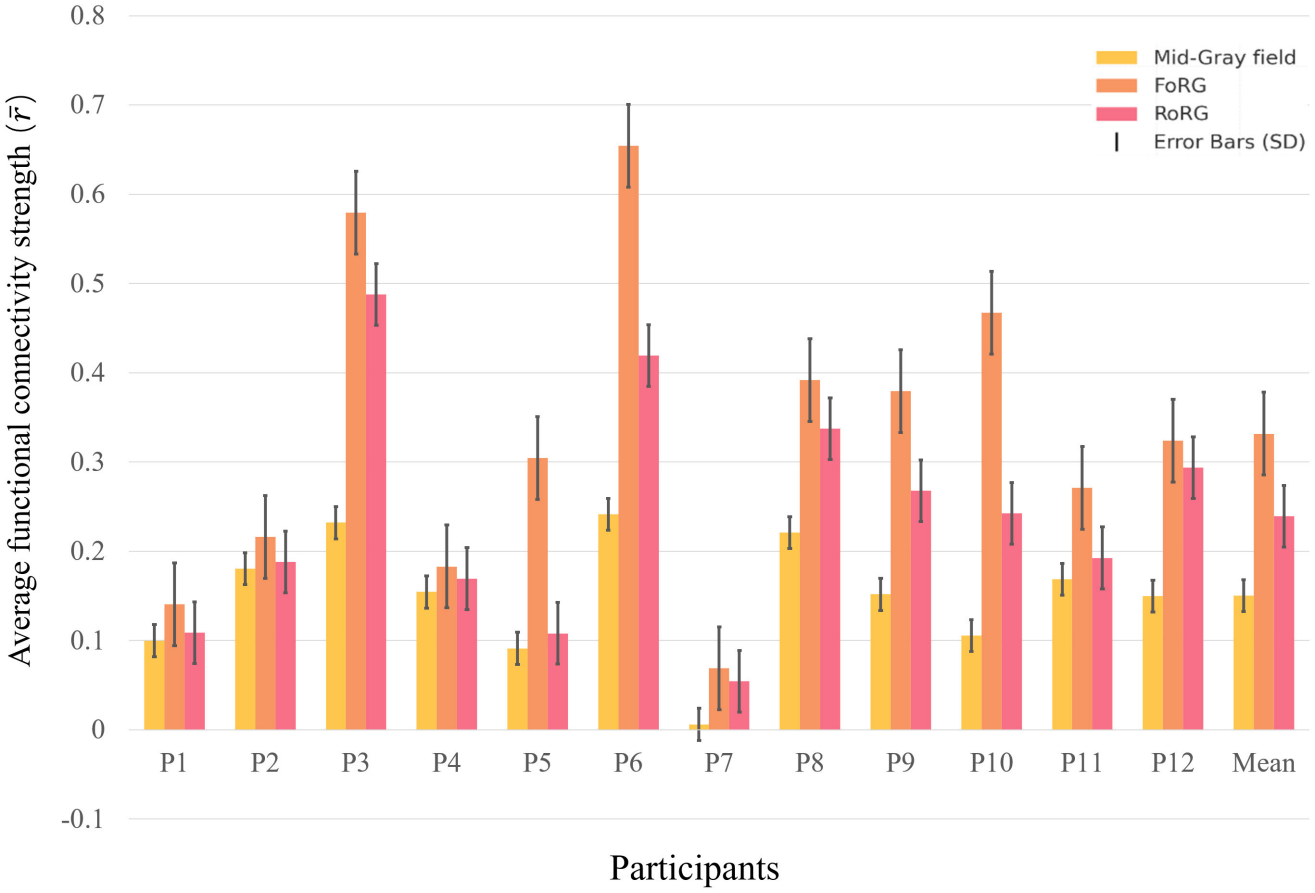
Mean brain functional connectivity strength for P1–P12. SD, standard deviation. Mid-Gray field = 0.0675, FoRG = 0.1746, RoRG = 0.1300.

Table 3 presents the mean brain functional connectivity strength values for each participant under the conditions of a Mid-Gray field image, binocular color fusion image, and binocular color rivalry image stimulation. Bold values indicate the mean brain functional connectivity strength under the FoRG image condition, and these bolded values represent the highest connectivity strength among the three conditions (Mid-Gray field, FoRG, and RoRG). Figure 7 presents each participant's mean brain functional connectivity strength using a bar chart. The data from Table 3, after calculation, yielded the following Standard Deviation (SD): Mid-Gray field = 0.0675, FoRG = 0.1746, RoRG = 0.1300. The data under all three conditions are relatively stable, with the Mid-Gray field condition showing the greatest stability.

**Table 3:** Mean functional connectivity strength values ($\bar{r}$) for each participant.

**Participants**	**Mid-gray field**	**FoRG**	**RoRG**
P1	0.0999	**0.1405**	0.1086
P2	0.1804	**0.2160**	0.1878
P3	0.2320	**0.5795**	0.4877
P4	0.1544	**0.1829**	0.1694
P5	0.0912	**0.3045**	0.1080
P6	0.2414	**0.6541**	0.4195
P7	0.0060	**0.0688**	0.0542
P8	0.2210	**0.3919**	0.3371
P9	0.1517	**0.3795**	0.2678
P10	0.1056	**0.4671**	0.2423
P11	0.1686	**0.2712**	0.1926
P12	0.1498	**0.3239**	0.2938
Mean	0.1502	**0.3317**	0.2391

Bold values represent the mean brain functional connectivity strength under the FoRG image condition, which is higher than the mean functional connectivity strength under the other two conditions (Mid-Gray field and RoRG).

In this study, the Friedman test was used to evaluate whether there were significant overall differences among the three conditions (Mid-Gray, FoRG and RoRG). The test results: Statistic ($\chi_{F}^{2}$) = 24, *P*-value=0.00001, indicating that there were statistically significant differences among the three conditions overall. Additionally, the *post-hoc* statistical power of the Friedman test was calculated to be 0.88, demonstrating that the experiment had a high power and was effective in detecting significant differences among the conditions. We further conducted pairwise comparisons among the three conditions using the Wilcoxon Signed-Rank Test with FDR correction. The results are as follows: (1) FoRG and RoRG: Statistic = 0, corrected *p*-value = 0.00049, indicating a significant difference. (2) Mid-Gray Field and FoRG: Statistic = 0, corrected *p*-value = 0.00049, indicating a significant difference. (3) Mid-Gray Field and RoRG: Statistic = 0, corrected *p*-value = 0.00049, indicating a significant difference. In the Wilcoxon Signed-Rank Test, a statistic of 0 means that the differences in the data are entirely in one direction, with no differences in the opposite direction. This indicates that the group differences are very strong and consistent.

Binocular color visual stimulation involves two primary processing modes: binocular color fusion and binocular color rivalry. These stimuli require coordinated cooperation between both eyes to integrate or compete with color information, resulting in complex visual experiences. This process engages multiple brain regions working in collaboration, and the increase in functional connectivity strength reflects the brain's efficient coordination in processing complex binocular visual information. Experimental results demonstrate that functional connectivity strength under binocular color visual stimulation conditions is significantly higher than under the Mid-Gray field condition. This indicates that in visual tasks requiring binocular coordination to process color information, the brain's functional connectivity becomes more active and robust. The Mid-Gray field represents a low-stimulation or basic visual state. Its functional connectivity strength is relatively low, reflecting the baseline activity level of the brain in the absence of specific visual tasks. Further experimental results reveal that the mean functional connectivity strength is highest under binocular color fusion, followed by binocular color rivalry, and lowest under the Mid-Gray field condition. This finding indicates that binocular color visual stimulation significantly enhances collaboration and integration between brain regions. Specifically, binocular color fusion exhibits a more pronounced increase in functional connectivity strength compared to binocular color rivalry, suggesting that the fusion process better facilitates cooperation and information integration among brain regions.

Additionally, the experiment observed considerable individual differences in functional connectivity strength under each condition. For example, P6 demonstrated the highest functional connectivity strength under both binocular color fusion and the Mid-Gray field conditions, while P7 exhibited lower connectivity strength across all conditions. These differences may be attributed to variations in participants' brain structure, functional state, or sensitivity to the stimuli. Despite these individual differences, the overall trend was consistent: all participants exhibited the highest mean functional connectivity strength under binocular color fusion stimulation and the lowest under the Mid-Gray field condition. This finding further highlights that binocular color fusion stimulation can significantly activate inter-regional cooperation in the brain, enhancing its ability to process complex visual information.

### 3.2 Reaction time analysis

**Figure 8:**
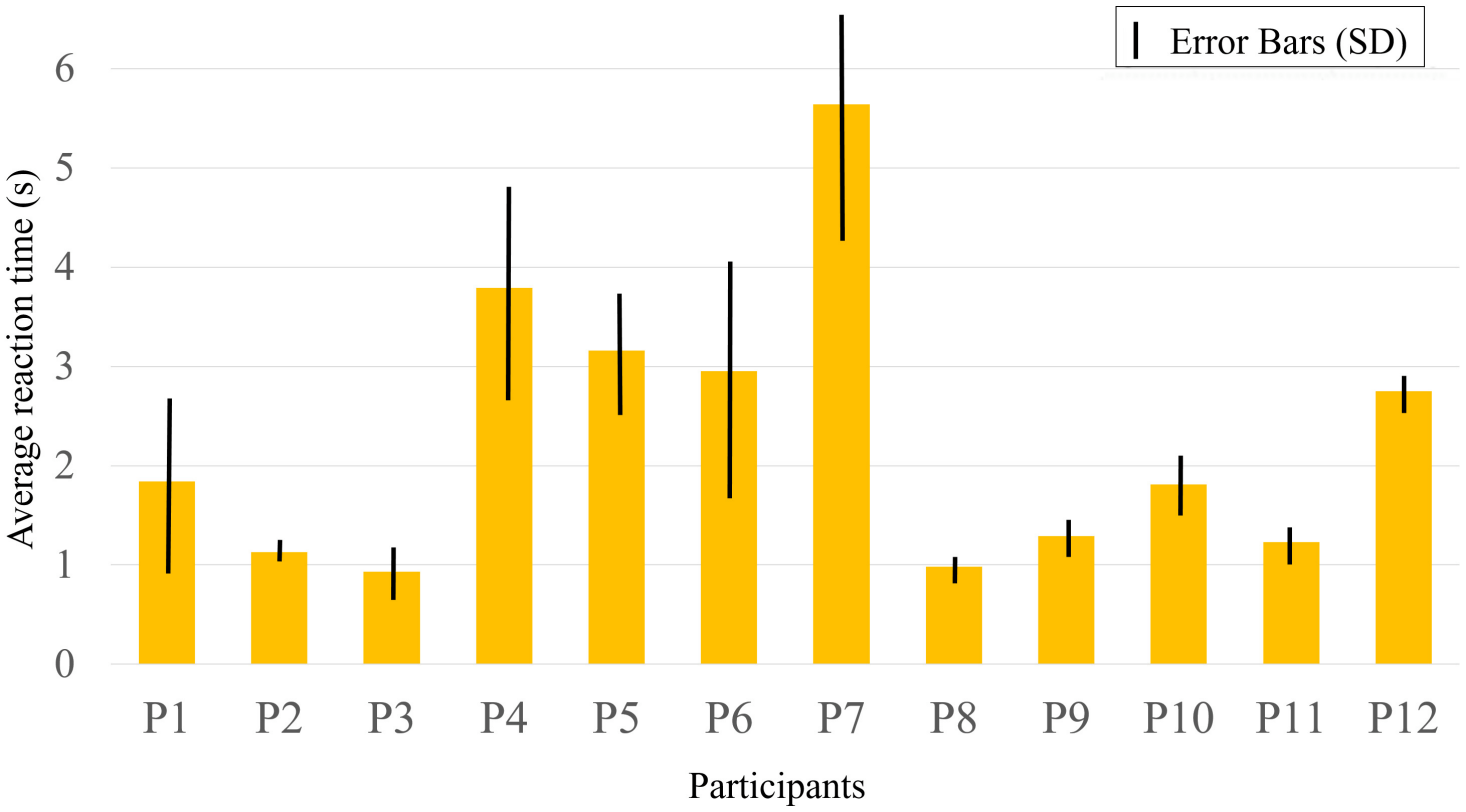
Mean reaction time performance for participants. The figure presents the mean reaction time of each participant along with its variability (standard deviation). The black vertical lines represent the standard deviation (SD), indicating the range of variability in reaction time for each participant.

This study analyzes the behavioral data (reaction time) collected from Experiment 2. Reaction Time (RT) was calculated as the difference between the time of the stimulus image presentation and the time when the “A” key was pressed. All participants made accurate judgments, responding only during the presentation of RoRG stimuli and not during the presentation of FoRG stimuli. This demonstrates that the subjective experiences elicited by binocular color rivalry and fusion are fundamentally different, and there is no mutual transformation between the two. Table 4 presents the reaction times, mean reaction times, and standard deviations for each participant in response to eight random binocular color rivalry image stimuli in a single experiment. Figure 8 visually illustrates the mean reaction times and standard deviations for participants responding to binocular color rivalry stimuli. Despite some individual differences, all participants' responses met the experimental requirements.

**Table 4:** Reaction times of participants in response to eight RoRG stimuli.

**Participants**	**RT (s)**								**Mean RT (s)**	**Standard deviation (SD)**
	**1**	**2**	**3**	**4**	**5**	**6**	**7**	**8**		
P1	6.5	1.0	2.5	1.1	0.9	0.8	0.8	1.1	**1.84**	1.96
P2	1.2	1.0	1.0	1.2	1.3	1.2	1.1	1.0	**1.13**	0.12
P3	0.8	0.7	0.7	1.7	0.9	1.0	0.7	0.9	**0.93**	0.33
P4	8.7	3.9	3.4	2.8	2.0	4.0	2.4	3.1	**3.79**	2.10
P5	2.7	2.2	5.6	2.8	4.4	2.5	2.8	2.3	**3.16**	1.20
P6	7.4	3.4	1.8	0.9	1.4	3.0	4.7	1.0	**2.95**	2.23
P7	2.9	6.8	3.6	8.6	5.9	7.5	6.8	3.0	**5.64**	2.19
P8	1.3	0.9	0.8	1.0	0.8	0.9	1.1	1.0	**0.98**	0.17
P9	1.5	1.2	1.3	1.1	1.0	1.8	1.4	1.0	**1.29**	0.25
P10	2.4	1.3	1.5	1.0	1.9	2.0	1.8	2.6	**1.81**	0.43
P11	1.2	1.1	1.2	1.0	1.5	0.9	1.3	1.6	**1.23**	0.25
P12	3.2	3.0	2.8	2.5	2.6	2.5	2.8	2.6	**2.75**	0.23

Bold values represents the mean reaction time across eight trials.

All participants' reaction times fell within the 10-s window of stimulus presentation. This observation indicates that all participants were able to accurately perceive the binocular color rivalry stimuli within the designated timeframe. It also confirms that the subjective experiences of binocular fusion and rivalry stimuli are distinct, enabling participants to make accurate judgments. P4 and P7 exhibited longer mean reaction times, ~3.79 s and 5.64 s, respectively. This may indicate slower responses during the task, potentially due to differences in color sensitivity, attention, or perceptual factors. In contrast, P3, P2, and P8 demonstrated shorter mean reaction times, close to 1 s, suggesting higher levels of focus and color sensitivity. All participants completed the task accurately, demonstrating their ability to effectively distinguish between the subjective experiences of binocular color fusion and rivalry stimuli. This result indicates that these two types of stimuli elicit entirely different perceptual experiences, enabling participants to make clear and precise judgments. The ability to differentiate between these conditions shows that the experimental design successfully presented robust and distinguishable visual stimuli, further validating the reliability of the experimental methodology and the robustness of the results. This distinction between binocular color fusion and rivalry stimuli proves that the perceptual and cognitive mechanisms involved in processing these two types of conditions are fundamentally different.

### 3.3 Regional analysis

**Figure 9:**
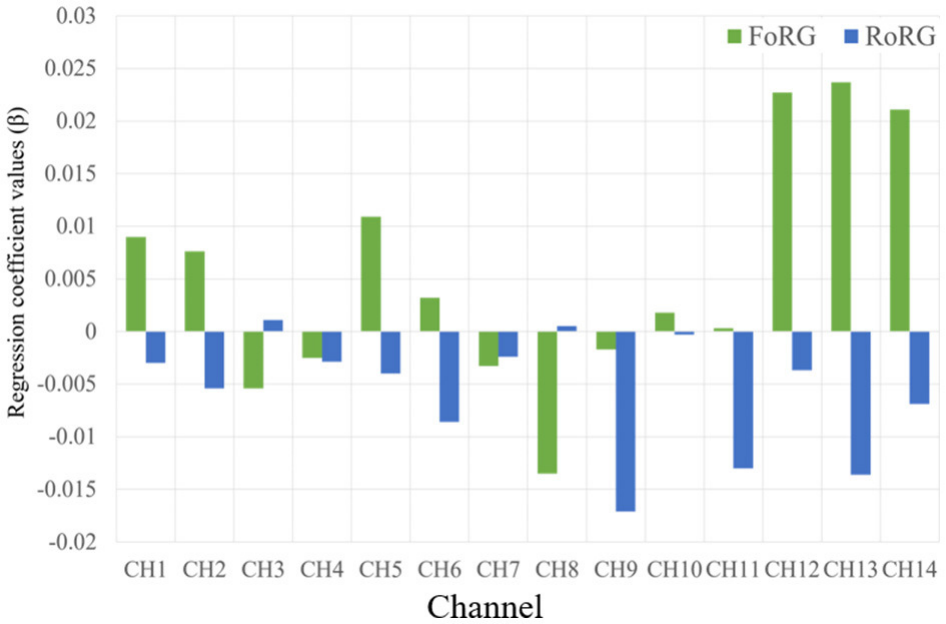
The mean β-value of FoRG and RoRG for CH1–CH14.

**Figure 10:**
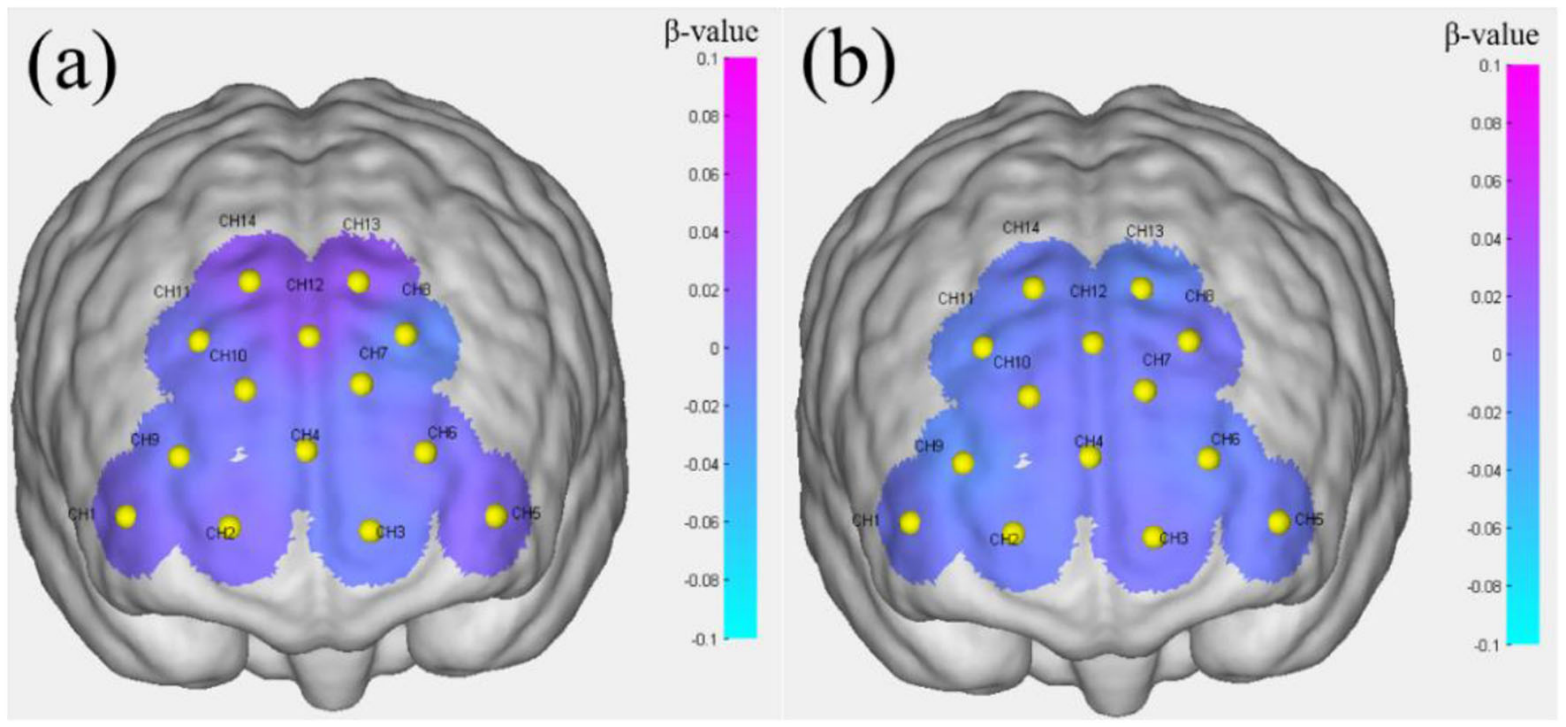
Brain activation levels. **(a)** Performance under binocular color fusion stimulation. **(b)** Performance under binocular color rivalry stimulation.

Table 5 presents the brain activation analysis results (β-value) for FoRG, while Table 6 provides the brain activation analysis results (β-value) for RoRG. Based on Tables 5, 6, Figures 8, 9 were created. Figure 9 shows the mean β-value across 14 channels under the two conditions, Through Figure 9, it is clearly evident that FoRG induces more neural activation in the prefrontal cortex compared to RoRG. Figure 10 presents the level of brain activation following the GLM analysis. Figure 10a shows the results under the binocular color fusion condition, where a large proportion of the area is shaded in purple, indicating high levels of brain activation. Figure 10b displays the results under the binocular color rivalry condition, where most areas are shown in blue, suggesting relatively low levels of brain activation. The higher level of activation under binocular color fusion, indicated by more extensive purple regions, suggests that the brain actively integrates the visual inputs from both eyes, which is a complex perceptual task. Conversely, the lower activation level under binocular color rivalry, predominantly shown by blue regions, indicates that fewer brain areas are activated. It implies that the brain processes a relatively more straightforward task with lower activation demands during the rivalry condition.

**Table 5:** Results of GLM analysis under FoRG.

**Channels**	**Regression coefficient values (**β**)**												
	**P1**	**P2**	**P3**	**P4**	**P5**	**P6**	**P7**	**P8**	**P9**	**P10**	**P11**	**P12**	**Mean**
CH1	−0.0210	0.1377	−0.0260	0.0237	−0.0139	−0.0131	−0.0200	0.0608	−0.0001	−0.0263	0.0119	−0.0052	**0.0090**
CH2	0.0024	0.1003	−0.0726	0.0285	0.0023	−0.0118	−0.0335	0.0421	0.0223	0.0033	−0.0046	0.0130	**0.0076**
CH3	−0.0339	0.0924	−0.0601	−0.0018	−0.0118	−0.0050	−0.0843	0.0582	−0.0065	−0.0452	0.0138	0.0201	**−0.0054**
CH4	−0.0226	0.0403	−0.0101	0.0187	−0.0091	−0.0096	−0.0538	0.0340	0.0558	−0.0690	−0.0155	0.0105	**−0.0025**
CH5	−0.0131	0.0751	−0.0109	−0.0151	−0.0111	−0.0164	0.0237	0.0650	−0.0462	−0.0171	0.0137	0.0830	**0.0109**
CH6	−0.0211	0.0509	−0.0026	0.0407	−0.0226	0.0101	−0.0573	0.0239	−0.0047	−0.0707	0.0109	0.0810	**0.0032**
CH7	−0.0128	0.0414	−0.0377	0.0354	−0.0195	−0.0141	−0.0137	0.0448	−0.0007	−0.0765	0.0062	0.0081	**−0.0033**
CH8	−0.0286	0.0358	−0.0368	0.0242	−0.0709	0.0265	−0.0505	−0.0057	−0.0073	−0.0745	0.0159	0.0103	**−0.0135**
CH9	−0.0170	0.1274	−0.0963	0.0122	−0.0184	0.0048	0.0740	−0.0039	−0.0681	−0.0566	0.0017	0.0196	**−0.0017**
CH10	−0.0020	0.0711	−0.0392	0.0089	−0.0014	−0.0033	0.0038	0.0462	0.0022	−0.0851	−0.0036	0.0244	**0.0018**
CH11	−0.0346	0.0613	−0.0411	0.0143	0.0069	−0.0013	−0.0022	0.0259	−0.0102	−0.0148	0.0152	−0.0163	**0.0003**
CH12	0.0007	0.0354	0.0282	0.0350	0.0015	0.0104	−0.0057	0.0527	0.0351	0.0638	0.0100	0.0055	**0.0227**
CH13	0.0045	0.0591	0.0392	0.0128	0.0005	0.0283	−0.0001	0.0437	0.0119	0.0297	0.0301	0.0247	**0.0237**
CH14	0.0087	0.0412	0.0036	0.0063	0.0099	0.0004	0.0254	0.0238	0.0451	0.0511	0.0289	0.0085	**0.0211**

Bold values represent the mean value of all participants for the channel.

**Table 6:** Results of GLM analysis under RoRG.

**Channels**	**Regression coefficient values (**β**)**												
	**P1**	**P2**	**P3**	**P4**	**P5**	**P6**	**P7**	**P8**	**P9**	**P10**	**P11**	**P12**	**Mean**
CH1	0.0293	−0.1053	−0.0245	0.0114	0.0237	−0.0352	0.0019	−0.0115	0.0181	0.0267	−0.0075	0.0372	**−0.0030**
CH2	−0.0106	−0.1013	0.0042	0.0056	−0.0003	−0.0061	0.0416	−0.0202	0.0270	−0.0520	0.0085	0.0389	**−0.0054**
CH3	−0.0117	−0.0883	0.0179	0.0168	0.0264	−0.0099	0.0377	−0.0182	−0.0123	0.0011	0.0081	0.0453	**0.0011**
CH4	−0.0228	−0.0437	−0.0080	−0.0056	−0.0028	0.0085	−0.0176	−0.0326	0.0080	0.0693	−0.0014	0.0141	**−0.0029**
CH5	−0.0412	−0.1357	−0.0242	0.0809	−0.0102	−0.0145	0.0895	−0.0106	−0.0149	0.0627	−0.0269	−0.0025	**−0.0040**
CH6	−0.0051	−0.0889	−0.0425	0.0153	−0.0266	−0.0216	0.0102	−0.0523	0.0000	0.0913	−0.0003	0.0172	**−0.0086**
CH7	−0.0099	−0.0296	−0.0071	−0.0141	−0.0201	−0.0171	0.0323	−0.0429	−0.0069	0.0935	−0.0147	0.0082	**−0.0024**
CH8	0.0126	−0.0396	0.0013	−0.0138	0.0954	−0.0668	0.0138	−0.0030	−0.0055	0.0493	−0.0135	−0.0242	**0.0005**
CH9	−0.0398	−0.0833	0.0202	−0.0141	−0.0338	−0.0173	−0.0230	−0.0010	0.0035	0.0291	−0.0169	−0.0286	**−0.0171**
CH10	−0.0031	−0.0394	0.0062	0.0061	−0.0125	−0.0129	0.0257	−0.0342	−0.0024	0.0425	0.0044	0.0164	**−0.0003**
CH11	0.0011	−0.0592	−0.0283	−0.0032	−0.0133	−0.0272	0.0150	−0.0160	−0.0014	−0.0165	−0.0191	0.0117	**−0.0130**
CH12	−0.0312	−0.0206	−0.0050	−0.0203	−0.0094	−0.0236	−0.0160	−0.0214	0.0389	0.0713	0.0010	−0.0086	**−0.0037**
CH13	−0.0022	−0.0452	−0.0076	0.0028	−0.0099	−0.0360	0.0008	0.0028	−0.0155	−0.0370	−0.0092	−0.0070	**−0.0136**
CH14	−0.0043	−0.0193	−0.0132	0.0012	−0.0200	−0.0405	0.0090	−0.0171	−0.0039	0.0527	−0.0295	0.0021	**−0.0069**

Bold values represent the mean value of all participants for the channel.

**Figure 11:**
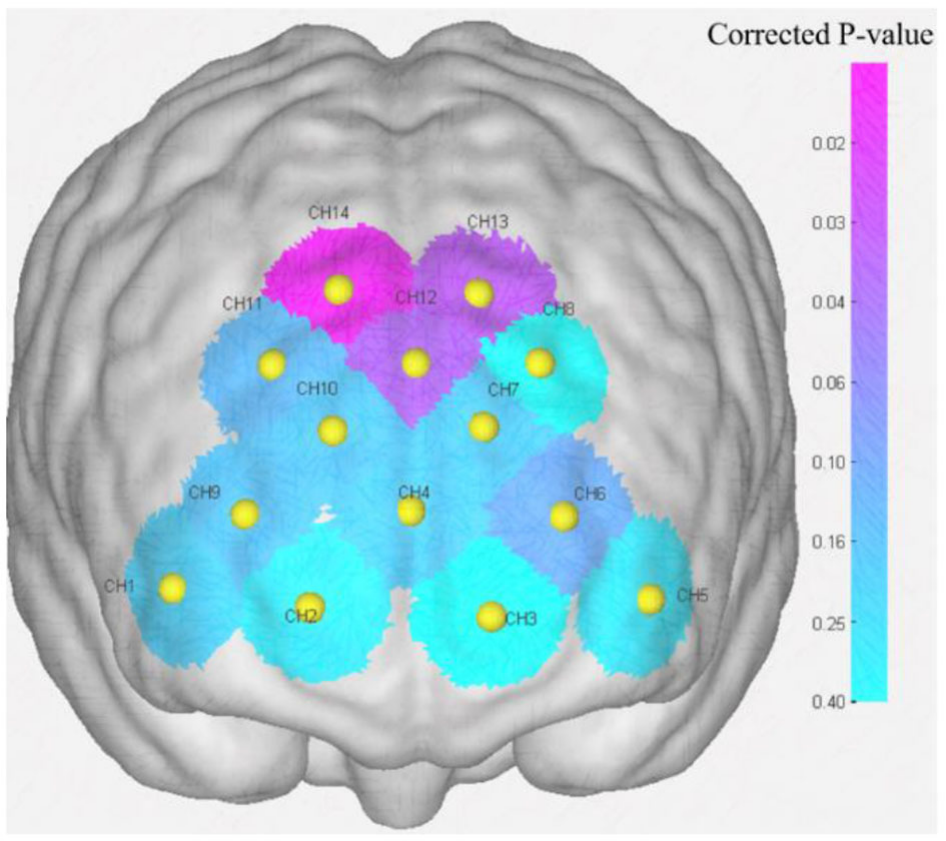
Corrected *P*-value representation of channels (CH12, CH13, and CH14: corrected *P* < 0.05).

After GLM analysis, the data under binocular color fusion and rivalry conditions were analyzed using the Wilcoxon Signed-Rank Test. Since both fusion and rivalry stimuli were tested on the same group of participants, the results for the two conditions are paired, making the Wilcoxon Signed-Rank Test an appropriate choice. This test is used to determine whether the two stimulation conditions have a significantly different impact on brain function. To ensure the accuracy of the test, the FDR correction method was applied. Table 7 presents the test statistic and corrected *P*-value for all channels, indicating the significance of the results for each channel. Among all the channels, CH12, CH13, and CH14 exhibit statistically significant differences (corrected *P* < 0.05), suggesting significant changes in brain activation between binocular color fusion and rivalry stimuli in these channels. Figure 11 shows that the corrected *P*-values for channels CH12, CH13, and CH14 are below 0.05, highlighting the notable activation differences in these channels under binocular color fusion vs. rivalry conditions. *Post-hoc* power analysis was conducted for the three channels with significant differences, and the results are as follows: (1) CH12: Effect size(*d*) = 1.09, Power = 0.92. (2) CH13: Effect size(*d*) = 1.28, Power = 0.98. (3) CH14: Effect size(*d*) = 1.35, Power = 0.98. The results of the power analysis indicate that the experimental design demonstrates high efficiency for these three channels. Given the current sample size and effect strength, the reliability of the test results is relatively high. These results are unlikely to be affected by insufficient sample size or inadequate experimental sensitivity.

**Table 7:** Statistic and corrected *P*-value for all channels.

**Channels**	**Statistic**	**Corrected *P*-value**	**Significant**
CH1	35	0.92285	No
CH2	38	0.96973	No
CH3	27	0.88563	No
CH4	38	0.96973	No
CH5	35	0.92285	No
CH6	27	0.88563	No
CH7	31	0.88563	No
CH8	31	0.88563	No
CH9	29	0.88563	No
CH10	33.5	0.92285	No
CH11	27	0.88563	No
CH12	3	0.01139	**Yes**
CH13	1	0.00684	**Yes**
CH14	1	0.00684	**Yes**

Bold values indicate significant results.

## 4 Discussion

Research indicates that binocular color fusion is a more challenging perceptual task than color competition, as it demonstrates stronger functional connectivity and greater neural activation in key brain regions. This suggests that color fusion requires more neural integration and cognitive control, demanding higher attention and executive processes compared to the relatively simpler task of color competition. Brain network analysis revealed that both binocular color fusion and competition stimuli significantly enhanced functional connectivity compared to a mid-gray field, with color fusion exhibiting the highest connectivity. This indicates that fusion demands greater cooperation among brain regions to integrate different visual inputs and form a unified perception. In contrast, binocular color competition only involves processing conflicting inputs, with lower integration demands.

Reaction time analysis showed that all participants responded accurately and consistently to RoRG stimuli, while no responses were observed for FoRG stimuli, suggesting that binocular color fusion and competition evoke entirely distinct subjective experiences. Further non-parametric tests of brain activation identified significant differences in three channels: CH12, CH13, and CH14. Among these, CH13 and CH14 are located in the Frontal Eye Fields (FEF), a region associated with visual attention and eye movement control. The significant differences suggest that binocular color fusion requires more eye movement and attention than competition. CH12 corresponds to the dorsolateral prefrontal cortex (DLPFC), a region responsible for working memory, decision-making, and cognitive control. Its activation further underscores the crucial role of the DLPFC in fusion tasks. General Linear Model (GLM) analysis supports this, showing that binocular color fusion involves a broader neural network and higher resource demands, including attention, perceptual integration, and cognitive control. These results highlight the greater neural demands of the fusion task compared to competition.

Research shows that binocular color fusion is a complex cognitive process that involves the collaboration of multiple brain regions. For instance, visual fusion requires the integration of visual information from both eyes, engaging the visual cortex and prefrontal cortex (29). This aligns with the enhanced functional connectivity found in this study, especially in the FEF and DLPFC, confirming their pivotal roles in the fusion process. Additionally, studies emphasize the importance of attention in visual integration, particularly in handling complex visual scenes (30, 31). The significant activation of the FEF during the fusion task further suggests widespread mobilization of attention resources. Our findings are consistent with these studies, demonstrating that binocular color fusion demands a higher degree of attention and cognitive control. Furthermore, research on binocular fusion mechanisms reveals that the brain must perform complex comparisons and matches of the two input signals, supporting the high cognitive demands of the fusion task (32). This helps explain the heightened neural activation observed under fusion conditions.

However, some studies present differing views on the difficulty and neural demands of binocular color fusion and competition. Blake and Logothetis (12) argue that while binocular competition has lower integration demands, it requires more inhibitory control and conflict management. They describe competition as a process where the brain continually suppresses input from one eye to ensure the dominance of the other. Other perspectives suggest that the duration and intensity of binocular competition are linked to an individual's cognitive control abilities, with stronger inhibitory functions potentially leading to significant activation in specific brain regions. This contrasts with the lower activation observed in our study under competition conditions (33). Additionally, binocular competition may involve active attention control and cognitive monitoring (34), which could explain the variation in brain activation levels during competition tasks.

In recent years, Brain-Computer Interface (BCI) research has made significant strides in decoding and utilizing brain responses to various visual stimuli (35, 36). The key regions identified in this study (CH12, CH13, and CH14) provide valuable insights into distinguishing binocular color fusion from competition, offering new directions for color-based BCI development. Binocular color fusion stimuli involve more complex perceptual integration, producing richer neural signals that may enhance the decoding of perceptual and attentional states. In contrast, neural responses to competition stimuli help us understand how the brain maintains selective attention while processing conflicting inputs (37). It is important to note that the study was conducted with a small sample size (12 participants), and small-sample studies can provide initial theoretical frameworks, exploratory findings, and methodological innovations. They help verify new technologies or theories, guide subsequent large-scale research, and offer a deeper analysis of specific issues under particular conditions. Additionally, the statistical methods used in this study were able to draw meaningful conclusions despite the small sample size. Furthermore, the experimental conditions may not fully capture the complexity of real-world visual environments, and individual differences in binocular fusion and competition have not been extensively studied, which may limit the generalizability of BCI applications (39). This study focused on the effects of visual stimuli on binocular fusion and rivalry but did not account for other sensory inputs. The fixed color settings, with the left eye always receiving green and the right always receiving red, may have introduced adaptation effects. Future research could employ randomized color presentations to explore functional connectivity and brain activation under varied conditions, providing a more comprehensive understanding of these complex visual cognitive processes.

## 5 Conclusion

This study integrates Experiment 1 and Experiment 2, utilizing brain network analysis and GLM methods to systematically examine the neural and behavioral differences between binocular color fusion and rivalry conditions. Non-parametric tests (Friedman Test and Wilcoxon Signed-Rank Test) were performed on the analysis results, with FDR correction applied to ensure the reliability of the statistical findings. Additionally, post hoc power analysis confirmed the appropriateness of selecting 12 participants, showing that their data were sufficiently robust to support the validity of this research.

Reaction time analysis revealed that binocular color fusion and rivalry elicit distinctly different subjective experiences. Participants were able to consistently and accurately distinguish between the two conditions, suggesting fundamental differences in their perceptual mechanisms. Further analysis revealed significant differences in brain connectivity and neural activation between binocular color fusion and rivalry, particularly in key regions (CH12, CH13, and CH14). CH12 is located in the DLPFC, while CH13 and CH14 are in the FEF. Compared to binocular color rivalry, binocular color fusion is a more demanding perceptual task that requires higher levels of neural integration and cognitive control. This is reflected in stronger functional connectivity and more pronounced neural activation in critical brain regions. The significant activation of the FEF and DLPFC further indicates that attention allocation and executive functions are vital in the integration process during fusion tasks. Visual fusion likely involves complex comparisons and matching of inputs from both eyes, increasing the demand for higher cognitive functions. The observed high functional connectivity suggests that the brain requires dynamic cooperation among multiple regions during fusion, reinforcing the idea that binocular fusion is a more complex perceptual task.

These findings offer key insights into the neural patterns that differentiate complex perceptual integration from simple conflict processing, deepening our understanding of the neural mechanisms underlying complex perceptual tasks. Future research could leverage these findings to develop personalized interventions or training strategies aimed at enhancing perceptual integration and cognitive control, particularly for individuals with weaker functional connectivity. Moreover, these results open new avenues for applying BCI technology. For example, in Virtual Reality (VR) and Augmented Reality (AR), BCI systems based on visual fusion signals could enhance user experience and reduce visual fatigue. In the medical field, such systems may be applied to diagnose and intervene in visual cognitive disorders, such as visual fatigue, attention deficits, or other neuro-related conditions. As technology evolves, BCI systems based on these findings are expected to become essential tools for future human-computer interaction and neurorehabilitation.

## Data Availability

The datasets presented in this study can be found in online repositories. The names of the repository/repositories and accession number(s) can be found below: https://doi.org/10.6084/m9.figshare.27330858.
